# Closed-Loop Adaptive Neurostimulation Technologies in Cognitive Rehabilitation of High-Tech Specialists

**DOI:** 10.17691/stm2022.14.4.04

**Published:** 2022-07-29

**Authors:** A.I. Fedotchev

**Affiliations:** Leading Researcher, Laboratory of Reception Mechanisms; Institute of Cell Biophysics of the Russian Academy of Sciences, 3 Institutskaya St., Pushchino, Moscow Region, 142290, Russia

**Keywords:** functional reliability and safety of a specialist, cognitive rehabilitation, non-invasive sensory stimulation, automatic modulation, human endogenous rhythms, stress-induced state correction

## Abstract

**The aim of the study** was to experimentally evaluate the applicability and effectiveness of two variants of the technology of adaptive neurostimulation with feedback from a person’s own rhythmic processes to increase the functional reliability and to reach cognitive rehabilitation of high-tech specialists.

**Materials and Methods:**

The study involved specialists who applied to the clinic with complaints of occupational pain syndromes and work stress. For the treatment of pain syndromes, analgesic electrical nerve stimulation was used with the parameters automatically modulated by feedback signals from the subject’s breathing rhythm. To correct stress-induced states, musical stimulation was used, automatically modulated by feedback signals from the narrow-band rhythmic components of the electroencephalogram (EEG) of the subject — alpha EEG oscillators. Treatment procedures without feedback from rhythmic processes were used as а control.

**Results:**

In the control sessions without the feedback from human rhythmic processes, no significant effects of stimulation were noted. With electrical stimulation controlled by the patient’s breathing (experiment 1), the most significant changes were observed in subjective pain scores, which dropped by half. A significant increase was noted in the power of the EEG alpha rhythm, respiration amplitude, and subjective ratings of well-being and mood. With music stimulation automatically modulated by the rhythmic components of the patient’s EEG (experiment 2), there was a significant increase in the power of the EEG alpha rhythm, as well as a decrease in the level of emotional disadaptation and stress.

**Conclusion:**

The data obtained clearly indicate that the developed and tested technologies of adaptive neurostimulation can be used for the timely correction of the functional state and cognitive rehabilitation of high-tech specialists by effectively eliminating the risks of their functional reliability caused by occupational pain and stress.

## Introduction

Human–machine interaction is a rapidly expanding field with a great need for the involvement of human factors in research and design [1]. One of the most important directions of modern human factors research is the problem of human operator reliability, which plays an important role in human–automation interaction in complex systems operation [2]. Human operator reliability includes at least three aspects: personal, professional, and functional reliability of a specialist [3]. Functional specialist reliability is related to the risks of operator’s failure or error as a result of a number of psychological and physiological conditions of the operators [4]. The most frequent risk-provoking conditions seem to be pain syndromes [5, 6], musculoskeletal disorders [7, 8], and stress-induced states [9, 10]. In the course of modern technological revolution, human operators are exposed to health and safety risks intrinsically related to automated tools and to greater psychosocial stress. In order to face these emerging risks, it is necessary to introduce specific practical, preventive and protective measures [11].

One of the possible solutions to this problem can be found by analyzing the trends in neuroergonomics, related to the creation and design of neurointerface systems that are better adapted to, and make use of the human information processing structures, including the body and the brain [12]. In particular, to reduce work-related risks of specialist’s reliability and safety and to reach cognitive rehabilitation, the methodology of closed-loop adaptive neurostimulation on-line computer-modulated by the rhythms of human brain and body could be successfully used. The advantages of using automatic closed-loop feedback from human endogenous rhythms in non-invasive adaptive neurostimulation procedures have been demonstrated in recent studies [13, 14].

Previously, two original versions of closed-loop adaptive neurostimulation have been developed and tested in two pilot studies. In the first one [15], analgesic electroneurostimulation with automatic modulation of the parameters of the stimulating current by the patient’s breathing rate was used in volunteers suffering from pains of different etiology. It was shown that, after only a single treatment procedure, the subjective pain ratings are significantly reduced. In another study [16], an original version of music therapy named “Music of the Brain” was applied for human health protection. This technique uses presentations of music or music-like stimuli on-line computer-modulated by the feedback from discrete components of the subject’s electroencephalogram (EEG) — EEG oscillators. It was shown that the presentation of music automatically modulated by the EEG oscillators of the patient leads to a decrease in the stress level, normalization of the EEG, and positive shifts in the psycho-emotional status of human subjects.

Despite the positive results of the pilot studies described, their significance is limited by the lack of control conditions. In order to assess the effectiveness of the developed approaches, it is necessary to compare the effects of the same kind of stimulation applied with and without a feedback from the subject’s rhythmical processes. Besides, it is still unclear if and how the methods of closed-loop adaptive neurostimulation can be applied to eliminate the risks of specialist reliability.

**The aim of the study** was to experimentally evaluate the applicability and effectiveness of two variants of the technology of adaptive neurostimulation with feedback from a person’s own rhythmic processes to eliminate the risks of specialist’s functional reliability and safety induced by work-related pain and stress.

For this, two experiments have been carried out, in which the participants were exposed in a random order to the same kind of stimulation applied with or without feedback from the subject’s rhythmical processes. Experiment 1 is aimed at assessing the effectiveness of electroneurostimulation automatically modulated by feedback signals from the patient’s breathing rate to eliminate work-related pain syndromes. Experiment 2 is aimed at assessing the effectiveness of music stimulation automatically modulated by the EEG oscillators of the patient to correct functional disturbances induced by stress.

## Experiment 1

### Materials and Methods

**Figure 1.**
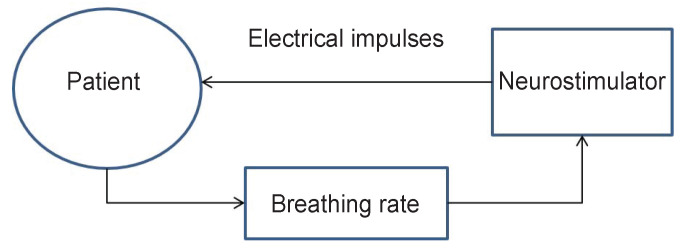
Key elements of electroneurostimulation modulated by patient’s breathing rate

In this experiment, high-tech professionals suffering from various pain syndromes participated in two treatment sessions, alternating in a random order. In one of two sessions (experimental), they were exposed to analgesic transcutaneous electroneurostimulation with the intensity on-line modulated by the subject’s breathing rate (Figure 1). In the other session (control), the same stimulation was applied with constant intensity without respiratory feedback.

***Participants*** — 14 patients of the Hospital of the Pushchino Scientific Center, Russian Academy of Sciences (5 women and 9 men aged from 35 to 62 years). These were responsible professionals involved in high-tech kinds of activity: programmers, PC operators, system administrators, and passenger bus drivers. They complained of occupational pain syndromes (stress headaches, neck pain, and pain in the wrist). The participants were informed about the research aims and methodology and were asked to sign the written consent form. The informed consent forms and all materials of the study were approved by the Ethics Committee of Institute of Cell Biophysics (Pushchino, Russia). The research was conducted in accordance with the Helsinki Declaration (2013).

#### Questionnaire

At the beginning and at the end of each examination, the level of pain has been evaluated for each patient using a standard visual analogue scale (VAS) of self-assessment of pain sensations on a 10-point scale [17]. Psychological testing of subjects was conducted using the standard Russian test "HAM" (health–activity–mood). This test allows a subject to assess the current state of his own health, activity, and mood. The test involved marking the points from 1 to 7 in 30 presentations on the proposed form, 10 for each of three scales. The numbers of points for each scale were the indicators of health, activity, and mood [18].

#### Registration of physiological data

After the initial psychological testing, subjects were installed with sensors for recording the electrophysiological characteristics, as well as with a breath sensor and stimulating electrodes located near the source of maximal pain sensations. A resistive respiration sensor fixed on the subject’s chest was used to register subject’s breathing. Then, a 2-minute recording of background electrophysiological characteristics (EEG and respiration pattern) was made. Monopolar EEG was recorded from the left occipital lead (point O1 by the international system 10–20%) with a combined ear reference. EEGs were amplified and digitized using a multichannel EEG amplifier Brainsys (Hardsoft, Russia) at a sampling rate of 128 Hz, power-line notch-filtered at 50 Hz, and band-pass filtered at 0.5–60.0 Hz. Simultaneously with the EEG, subject’s respiration pattern was registered and processed.

#### Stimulation

After baseline recording, electrical stimulation was performed for 15 min both for experimental and control sessions. A serial transcutaneous electroneurostimulator ETNS-100-01 (Russia) with electrical pulses at a frequency of 4 Hz and a maximum intensity of 10 mA was used. During an experimental trial, the amplitude of electrical pulses was automatically modulated by the own respiratory rhythm of the patient within 0.1–10.0 mA due to inclusion of feedback signals from the subject’s breathing to the stimulator output circuit. The deeper was the subject’s inhalation, the more intensive was stimulation. During a control examination, the respiratory sensor was disconnected from the neurostimulator, and the stimulation intensity was constantly maintained at a middle level of 5 mA. After stimulation, electrographic indicators were repeatedly recorded.

#### Data analysis

The statistical processing of the results was carried out using the Sigma-Plot 11.0 software package. After passing the Shapiro–Wilk test of normality, repeated measures multiway ANOVA was used to assess the statistical significance of the shifts for each indicator after treatment. The post hoc test was applied using the Bonferroni correction (a 0.05 or 95% confidence interval) to find the difference between control and experimental sessions. The paired t-test was used to determine the mean values (M) and standard deviations (σ) for the shifts of the indicators after treatment relative to the background and to assess the significance levels of these shifts. The values were considered significant if p<0.05.

### Results

The effects in the control (without feedback) and the experimental (breathing-modulated electrostimulation) sessions were evaluated by comparing the shifts of main indicators as a result of the treatments (Table 1).

**Table 1:** Mean values of objective and subjective indicators shifts after treatment (experiment 1)

Indicators	Session			
	Control		Experiment	
	M±σ	p	M±σ	p
Alpha EEG power (relative units)	0.43±1.45	0.290	1.21±1.31	**0.004**
Breathing rate (cycles/min)	0.36±1.0	0.208	1.43±2.21	**0.031**
Breating amplitude (μV)	0.28±1.85	0.575	5.86±2.31	**0.001**
Evaluation of pain (points)	–0.21±0.58	0.189	–1.64±0.63	**0.001**
HAM test (points):				
health	1.21±2.70	0.116	1.86±0.77	**0.001**
activity	–0.43±1.40	0.272	0.21±0.97	0.426
mood	0.36±1.0	0.208	1.14±1.40	**0.009**

Note: p is statistical significance of value differences in relation to the basal value.

The data in Table 1 show that the treatment produces a significant effect only in the experimental conditions. Under the electrical stimulation modulated by the subject’s breathing, significant shifts are registered both for objective and subjective indicators. Among the significant changes in objective electrophysiological characteristics are the growth of alpha EEG power and the change in the nature of respiration which become more frequent and has increased amplitude. According to post-treatment interviews, the subjects began to breathe deeper to enhance the positive effects of analgesia.

Under electrical stimulation modulated by a patient’s breathing, the most significant changes were observed in subjective assessments of the pain level. A reduction in pain was accompanied by a significant increase in self-ratings of health and mood (HAM test).

### Discussion

Transcutaneous electrical nerve stimulation (TENS) is a non-pharmacological intervention that activates a complex neuronal network to mitigate pain by activating descending inhibitory systems in the central nervous system [19]. It seems now reasonable to use TENS as a pain management intervention [20]. However, the available TENS treatments have essential problems [21], and neuromodulatory interventions that modify brain processes underlying the experience of pain have a potential to provide substantial pain relief [22].

In our study, the most significant pain-reducing effects accompanied by significant changes in objective and subjective characteristics were registered under closed-loop adaptive neurostimulation with a patient’s breathing as a modulating factor. From the literature, it is known that breathing is a fundamental rhythm of brain function. Respiration, via multiple sensory pathways, modulates the temporal organization of cortical neurodynamics, thereby linking higher cortical functions to the process of breathing [23, 24]. Breathing can act as an organizing hierarchical principle for neuronal oscillations throughout the brain and detail mechanisms of how cognitive factors impact otherwise automatic neuronal processes during interoceptive attention [25].

These considerations permit us to suggest that, through the modulation of electrical stimulation amplitude by the patient’s own breathing, a resonance activation of the brain structures that receive TENS-produced signals and mediate pain management can occur. Using closed-loop adaptive electrostimulation automatically modulated by the patient’s breathing, it is possible to effectively eliminate the risks of specialist’s reliability and safety induced by work-related pain, and to implement the cognitive rehabilitation.

## Experiment 2

### Materials and Methods

**Figure 2.**
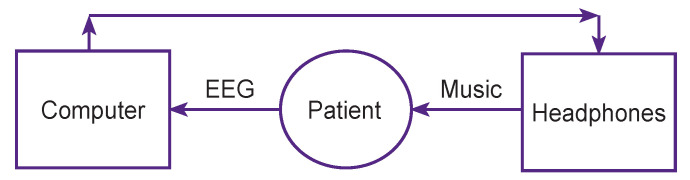
A scheme of correction of stress-induced disorders via musical stimulation controlled by the brain biopotentials of a patient

In this experiment, high-tech professionals suffering from work-related stress participated in two examinations. In one of the examinations (experimental), they were exposed to classical music with a loudness on-line modulated by the patient’s EEG oscillators (Figure 2). In another examination (control), the same music stimulation was applied without EEG feedback.

***Participants*** — 17 specialists, researchers and computer programmers (8 women, 9 men aged from 35 to 62 years). They complained of psycho-emotional tension and stress due to urgent workload and voluntarily agreed to participate in the treatment sessions. The participants were informed about the research aims and methodology and were instructed to sit quietly with closed eyes, listening to the sounds through headphones. The participants were asked to sign the written consent form. The informed consent forms and all materials of the study were approved by the Ethics Committee of Institute of Cell Biophysics (Pushchino, Russia). The research was conducted in accordance with the Helsinki Declaration (2013).

#### Questionnaire

At the beginning and at the end of each session, patients were psychologically tested using the three standard Russian tests described earlier [26]. In the HAM test, the participants were asked to perform a self-assessment of their current state of health, activity and mood by marking the points on a seven-point scale between 30 pairs of adjectives indicating the opposite estimates of three states (e.g., feeling well versus feeling sick, active versus passive, happy versus unhappy). The number of points accumulated for each state in 10 presentations indicated the current state of the subject’s health, activity, and mood.

In the emotional disadaptation (ED) test, the participants were presented a circle divided into 4 sectors. Within each sector, there were adjectives describing the emotional state of a person, corresponding to four basic personality requirements: safety, independence, achievement, unity–intimacy. The patients were asked to select the sector in the round that corresponded to their current state within three attempts. Each sector had its own score (from 1 to 4), which was not shown to the participant. The scored sum of points served as an indicator of the degree of emotional maladjustment of the person.

The stress level (SL) test was similar to the ED test, but the adjectives corresponded to the level of stress of a person.

#### EEG recording and analysis

Monopolar EEG was recorded from the left occipital lead (point O1 by the International system 10-20%) with a combined ear reference. EEGs were amplified and digitized using a multichannel EEG amplifier Brainsys (Hardsoft, Russia) at a sampling rate of 128 Hz, power-line notch-filtered at 50 Hz, and band-pass filtered at 0.5–60.0 Hz. During background EEG registration, the dominant eyes closed narrow frequency (0.6 Hz) EEG oscillator from the alpha (8 to 13 Hz) EEG band was identified in each participant. For this purpose, procedures of fast Fourier transform were performed for short (5 s) periods of background EEG, that were sequentially shifted relative to each other with 50% overlap. To increase the signal/noise ratio, during the histogram accumulation of the short-term spectra their local maxima only were taken into account with zero padding for the rest of spectrum.

When individual spectral peaks are sequentially accumulated for a 30-second period of background EEG recording, the resulting spectrum is based on the summation of 11 short-term spectra; it has 0.2 Hz frequency resolution and provides information on a narrow-band EEG oscillator (peak frequency ±0.2 Hz), that is stable and important for the individual [27]. The revealed alpha EEG oscillator of the individual was then used for the feedback control of music presentation.

#### Procedure

After the initial psychological testing, the subjects were equipped with EEG sensors using a special headset and stereo headphones (Philips SBC HL140, sound level 0–40 dB, frequency 100–2000 Hz). Then, EEG was recorded for 30 s to identify the alpha EEG oscillator in each subject. After baseline recording, EEG registration was continued, and the participants were presented classical music. In one of two treatment sessions, music was presented to the subject without EEG feedback (control session). In the other one, music loudness was modulated by the current amplitude of the subject’s alpha EEG oscillator (experimental session). To avoid artifacts, during both sessions the subjects were instructed to sit quietly with eyes closed and listen to musical excerpts. At the end of each experiment, the subjects were retested and interviewed about their feelings during the treatments.

#### Stimulation

Two types of stimulation were used for each subject in a counterbalanced order. In one session (control), the subjects were presented a 10-minute composition from popular classical music by Mozart, Bach, and Schubert, pre-recorded on the hard disk of the computer. The same audio file was used in the other session with a feedback from the subject’s EEG oscillator (musical EEG feedback). Owing to the specially developed software, the music sound intensity was modulated online by the integral amplitude of the alpha EEG oscillator of the individual: the bigger its amplitude, the louder the music, and vice versa.

#### Data analysis

The statistical processing of the results was carried out using the Sigma-Plot 11.0 software package. After passing the Shapiro–Wilk test of normality, repeated measures multiway ANOVA was used to assess the statistical significance of the shifts for each indicator after treatment. The post hoc test was applied using the Bonferroni correction (a 0.05 or 95% confidence interval) to find the difference between the control and experimental examinations. The paired t-test was used to determine the mean values (M) and standard deviations (σ) for the shifts of the indicators after treatment relative to the background and to assess the significance levels of these shifts. The values were considered significant if p<0.05. To determine the differences between the control and experimental conditions (control vs experimental), the Mann–Whitney rank sum test was used.

### Results

The effects in the control (listening to music without feedback) and experimental series (musical EEG feedback) were evaluated by comparing the shifts of the main indicators as a result of the treatments (Table 2).

**Table 2:** Mean values of objective and subjective indicators shifts after treatment (experiment 2)

Indicators	Session				p**
	Control		Experiment		
	M±σ	p*	M±σ	p*	
Alpha EEG power (relative units)	1.3±3.5	0.162	6.0±3.3	**0.001**	**0.001**
Theta EEG power (relative units)	–0.5±1.2	0.135	–0.3±1.7	0.493	0.396
HAM test (points):					
health	1.3±2.9	0.096	4.9±4.5	**0.001**	**0.015**
activity	–0.3±3.4	0.721	2.7±5.9	0.090	0.074
mood	0.7±2.6	0.270	5.5±5.8	**0.002**	**0.006**
ED test (scores)	–0.1±1.2	0.697	–1.0±1.2	**0.005**	**0.035**
SL test (scores)	–0.1±1.0	0.817	–1.0±1.0	**0.002**	**0.020**

Note: * statistical significance of values in relation to the basal value; ** between the control and experimental sessions.

The data in Table 2 show that the alpha EEG power increases, and theta EEG power decreases after both treatments. However, a significant increase was noted only in the experiments with a feedback from the EEG. The difference in the shifts of alpha EEG power between the control and experimental sessions was highly significant (p<0.001). It can also be seen that, as a result of therapeutic procedures with a feedback from the EEG, positive changes occur in the subjective indicators of health and mood in the HAM test. After both treatments, the levels of emotional disadaptation (ED test) and stress (SL test) of the subjects decreased. However, these changes have reached the level of significance only in the case of a feedback from the EEG.

The questioning of subjects about the sensations during the experiments revealed their positive attitude to the treatment sessions, a lowering in the level of stress, and an improvement of the emotional state.

### Discussion

It is known that music itself can be effectively used to change the psychophysiological status of humans [28, 29] and provides a framework for the development of non-pharmaceutical treatments of neurological disorders [30]. Some studies use music-based neurointerfaces to govern mental states and mediate mood disorders [31-33].

The present study was aimed at evaluating the efficiency of the EEG-based musical neurointerface for the elimination of stress-induced functional disturbances. For this, the effects of simple listening to prerecorded classical music were compared with the effects observed in the experiment where the same music is presented in strict accordance with the current amplitude of the alpha EEG oscillator of an individual.

A significant increase in the indicators was noted only in the experimental condition (experiment) with a feedback from the EEG. Based on the literature data [34, 35], this result can be considered as an indicator of a wakefully relaxed state and internalized attention induced by the EEG-modulated musical intervention. From the literature, it is also known that the fluctuations in the electrical activity of the brain can be synchronized with the temporal patterns of external influences and lead to the therapeutic effect of music [36]. The therapeutic effects of the EEG-based musical intervention are evidenced by significant positive changes in the indicators of health and mood of the subjects after the treatment, as well as by a significant decrease in the level of emotional disadaptation and stress.

The results obtained are in line with the results of a recent study where the acoustic stimuli on-line generated by the software-guided transformation of the subject’s dominant EEG rhythms were used to decrease the post-traumatic stress symptoms [37]. The authors came to the conclusion that rapid updating regarding its own pattern, and resonance between the audible tones and oscillating brain networks, provides the brain a chance to auto-calibrate, self-adjust, “relax”, and reset/get unstuck from what have been persisting stress/trauma response patterns.

Enhanced efficiency of EEG-based musical neurointerface may be related to the presentation of music in strict accordance with the relevant brain bioelectric characteristics of the individual. An optimal condition for this is the utilization of a patient’s narrow-band oscillator from the alpha EEG range, e.g., the alpha EEG oscillator of the patient. As shown previously [38], in this case the conditions are created for optimal involvement of integrative, adaptive, and resonance mechanisms of the central nervous system into complex organism’s reactions to low-intensity environmental influences.

In conclusion, the results obtained suggest the possibility of successful use of the EEG-based musical neurointerfaces in a wide range of cognitive rehabilitation procedures, including the elimination of the risks of specialist reliability and safety induced by stress.

## General discussion of experiments

The results of our study clearly demonstrate the advantages of the closed-loop adaptive neurostimulation approach, where sensory stimulation is automatically adjusted in response to dynamic changes in the person’s own rhythmical activity. In particular, experiment 1 shows that, under analgesic transcutaneous electroneurostimulation on-line modulated by the patient’s breathing rate, the level of subjective pain ratings dropped by half after only a single treatment procedure. Experiment 2 shows that, with the presentation of music automatically modulated by a feedback from the patient’s EEG oscillators, a decrease in the stress level, a normalization of the EEG, and positive shifts in the psycho-emotional status of human subjects are observed.

The results obtained can be explained by the main peculiarity of the developed approach that is related to the automatic modulation of sensory stimulation by the human body rhythmic processes. These rhythmical processes are closely interrelated [23] and form the basis for homeostatic constancy, efficiency of physiological processes, and the adaptation to internal/external changes and requirements [39]. Endogenous body rhythms participate in rhythmic facilitation of sensory processing [40] and are a source of interoceptive signals that play an important role in maintaining the optimal physical, emotional and mental health of a person [41].

## Conclusion

Automatic modulation of sensory stimulation by the human endogenous rhythms has a number of advantages, such as the dynamic nature and high personalization of therapeutic procedures, the involvement of interoceptive signals in the mechanisms of multisensory integration, neuroplasticity and resonance mechanisms of the brain, and the automatic, without conscious efforts of a person, control of therapeutic sensory stimulation. The results obtained clearly indicate that human body rhythms — the respiratory rate and EEG rhythms — can be successfully used to eliminate the risks of specialist’s reliability and safety induced by pain and stress.
